# Is Handwriting Performance Affected by the Writing Surface? Comparing Preschoolers', Second Graders', and Adults' Writing Performance on a Tablet vs. Paper

**DOI:** 10.3389/fpsyg.2016.01308

**Published:** 2016-09-12

**Authors:** Sabrina Gerth, Annegret Klassert, Thomas Dolk, Michael Fliesser, Martin H. Fischer, Guido Nottbusch, Julia Festman

**Affiliations:** ^1^Research Group: Diversity and Inclusion, Human Sciences Faculty, University of PotsdamPotsdam, Germany; ^2^Department of Psychology, University of RegensburgRegensburg, Germany; ^3^Cognitive Sciences, University of PotsdamPotsdam, Germany; ^4^Primary School Education/German, Human Sciences Faculty, University of PotsdamPotsdam, Germany

**Keywords:** handwriting, movement kinematics, writing acquisition, children, graphomotor control, tablet

## Abstract

Due to their multifunctionality, tablets offer tremendous advantages for research on handwriting dynamics or for interactive use of learning apps in schools. Further, the widespread use of tablet computers has had a great impact on handwriting in the current generation. But, is it advisable to teach how to write and to assess handwriting in pre- and primary schoolchildren on tablets rather than on paper? Since handwriting is not automatized before the age of 10 years, children's handwriting movements require graphomotor and visual feedback as well as permanent control of movement execution during handwriting. Modifications in writing conditions, for instance the smoother writing surface of a tablet, might influence handwriting performance in general and in particular those of non-automatized beginning writers. In order to investigate how handwriting performance is affected by a difference in friction of the writing surface, we recruited three groups with varying levels of handwriting automaticity: 25 preschoolers, 27 second graders, and 25 adults. We administered three tasks measuring graphomotor abilities, visuomotor abilities, and handwriting performance (only second graders and adults). We evaluated two aspects of handwriting performance: the *handwriting quality* with a visual score and the *handwriting dynamics* using online handwriting measures [e.g., writing duration, writing velocity, strokes and number of inversions in velocity (NIV)]. In particular, NIVs which describe the number of velocity peaks during handwriting are directly related to the level of handwriting automaticity. In general, we found differences between writing on paper compared to the tablet. These differences were partly task-dependent. The comparison between tablet and paper revealed a faster writing velocity for all groups and all tasks on the tablet which indicates that all participants—even the experienced writers—were influenced by the lower friction of the tablet surface. Our results for the group-comparison show advancing levels in handwriting automaticity from preschoolers to second graders to adults, which confirms that our method depicts handwriting performance in groups with varying degrees of handwriting automaticity. We conclude that the smoother tablet surface requires additional control of handwriting movements and therefore might present an additional challenge for learners of handwriting.

## Introduction

The rapid technological developments and advanced digitization in all aspects of human life require research to assess the significance of how to impart knowledge to students via these new media. When students enter school today they are already members of the generation known as *digital natives* (Chicu et al., 2014). They understand how to use computers to quickly find and assimilate new information. The teacher's challenge is to use the technology and help students in mastering new subjects in a creative, autonomous, critical, and communicative way. Nevertheless, new technologies such as tablets are currently only selectively used in schools (at least in Germany) as revealed by the International Computer and Information Literacy Study in 2013 (Bos et al., 2014). The results of the ICILS show that only 6.5% of eighth graders in Germany attend a school that uses tablets for teaching purposes (EU average: 15.9%; Australia: 63.6%). Should the answer to this low percentage be to blindly introduce tablets to schools? Or is there a need to assess specific advantages and disadvantages of tablet use before their introduction? In support of the latter, the purpose of our study was to investigate whether it makes a difference for beginning learners (preschoolers and second graders) to write on a tablet screen compared to on common paper. Further, we compared these results to those of experienced writers (adults) to explore how the use of tablets influences groups with different levels of handwriting abilities.

Handwriting requires the coordination of a complex and fine-tuned mechanism involving multiple muscles in the hands, arms, and even the shoulder (Latash, 1993; Huber and Headrick, 1999). Their precise interplay generates skilled and controlled movements with a writing instrument (e.g., a pen or a pencil). Writing involves the execution and combination of specific strokes in a particular sequence. Furthermore, to produce fluent writing movements one must constantly use visual monitoring and sensorimotor feedback (Fischer and Wendler, 1994; Tseng and Chow, 2000). Handwriting models are typically organized hierarchically (Flower and Hayes, 1981; Van Galen, 1991; Berninger et al., 1998). These models postulate that activities at lower levels (e.g., graphomotor planning and execution) interact with performance at higher levels (e.g., syntax, semantics, creation of ideas; Van Galen, 1991; Abbott and Berninger, 1993; Graham and Weintraub, 1996). As soon as lower level abilities are fully mastered and can be executed automatically, more resources become available for higher level processes. Research on early handwriting acquisition suggests that the coordination of perceptual, motor, and cognitive processes is critical for efficient and fluent handwriting movements (Maldarelli et al., 2015).

The development of handwriting abilities starts even before entering school and prior to formal writing instructions on how to write letters, words and sentences, for example when children practice drawing or scribbling (Gombert and Fayol, 1992; Fischer and Wendler, 1994; Adi-Japha and Freeman, 2001). Children need to visually distinguish forms and symbols to be able to reproduce them accurately (Fischer and Wendler, 1994). Research with typically developing children has shown that between the ages of 6 and 7 the quality of handwriting develops rapidly which coincides with the start of formal writing instructions at school (Feder and Majnemer, 2007). Before the age of 10 the children's handwriting movements are slow and require graphomotor and visual feedback, only around the age of 14 years writing movements become fast and automatic, which releases more resources for higher level processes of writing (Huber and Headrick, 1999; Chartrel and Vinter, 2006; Pontart et al., 2013). The acquisition of writing is accompanied by a decrease in conscious attention to and control of the graphomotor execution, thus leading to an automatization of the writing process.

Previous research comparing adults' and children's writing abilities revealed that less skilled writers exhibit longer pauses between writing units and use more strokes to produce letters (Rosenblum et al., 2003, 2006; Sumner et al., 2013; Kandel and Perret, 2014; Julius and Adi-Japha, 2015). Experienced writers are able to plan their writing movements in advance and execute them more smoothly (shorter the time that the pen spends on the writing surface), compared to less skilled writers who rely more often on in air times of the pen tip between writing units for planning (longer time when the pen is above the writing surface; Julius and Adi-Japha, 2015). In an intervention study Julius and Adi-Japha (2015) revealed that kindergarten children improved strongest when compared to second graders and adults for writing time and for in air time in a point-to-point connection task to produce a letter-like symbol. A second study, by Kandel and Perret (2014), showed that even children between 8 and 10 years, who are in the middle of handwriting acquisition, already use the ability of *motor anticipation* to write fast and smoothly. Motor anticipation refers to the ability to write one letter while already processing information on how to produce the next letter. Through writing practice the children generate so-called motor programs that contain information on how the letters are shaped and the exact number, order and direction of the respective strokes (Meulenbroek and Van Galen, 1989; Kandel and Perret, 2014). This consolidation process requires years of practice and learning. As soon as the writer is able to activate the motor programs quickly and effortlessly the handwriting movements become automatic, continuous, and fast (Kandel and Perret, 2014). In the Kandel and Perret (2014) study children had to write letter sequences (*ll, le*, and *ln*) in cursive handwriting on a digitizer. The movement time of the up- and down-strokes indicated that motor anticipation of letter size changes (*ll* vs. *le*) and directional changes (*le* vs. *ln*) helped to reduce dysfluencies which decreased from 8 to 9 years and remained stable between 9 and 10 years. Dysfluent movements were mostly observed for down-strokes, which might suggest that the writer anticipated the motor sequence of the next letter.

Handwriting abilities can be divided into different dimensions, namely graphomotor, visuomotor, and handwriting. Regarding graphomotor abilities, studies have shown that it seems to be easier for children to draw horizontal lines to indicate spatial axes (e.g., the sky, the ground) than drawing vertical lines denoting depth of objects (Lange-Küttner, 1998). Even more difficult than vertical lines are diagonal lines that children acquire only at around 7 years of age (Laszlo and Broderick, 1991). A study by Meulenbroek and Van Galen (1986) showed that children between 6 and 9 years drew repetitive loops with a shorter duration and a higher velocity compared to zigzag lines.

Another important aspect of handwriting are visuomotor abilities. Visual-motor integration refers to the interaction of visual skills, visual-perceptual skills, and motor skills (Exner, 2010) and is known to play a crucial role in handwriting acquisition (Weil and Cunningham-Amundson, 1994; Tseng and Chow, 2000; Daly et al., 2003; Volman et al., 2006; Kaiser et al., 2009). Significant correlations between the results of the developmental test of Visual-Motor Integration (VMI; Beery and Beery, 2010) and the quality of handwriting are found such that children who achieve a higher score in visuomotor tasks write faster (Tseng and Chow, 2000) and have a better handwriting quality (Weil and Cunningham-Amundson, 1994; Cornhill and Case-Smith, 1996). As soon as the child can accurately copy the first 9 forms of the VMI he or she is ready to acquire handwriting (Weil and Cunningham-Amundson, 1994). To assess handwriting abilities of adults and children, previous studies usually used the alphabet writing task or the firstname-surname task (Pontart et al., 2013; Alamargot and Morin, 2015). In the alphabet task participants had to write the alphabet in the correct order in lower-case letters (Abbott and Berninger, 1993). For the firstname-surname task participants must write their own name repeatedly. Both tasks are supposed to mirror highly automatized writing movements that directly reflect handwriting abilities. However, both tasks introduce uncontrolled between-participants variability, because the letters in the alphabet are not ordered according to complexity in number or direction of strokes, and first names or surnames differ in the number, complexity and frequency of letters (Tim vs. Samantha).

Regarding handwriting abilities, research has mostly focused on examining the product of writing. The quality of handwriting was evaluated as the accuracy of letter formation, the uniformity of letter size, the spacing between letters and words, and the alignment on lines of writing (Hamstra-Bletz and Blöte, 1993). The assessment of quality is usually done by copying words or a sentence (e.g., “the quick brown fox jumps over the lazy dog”) or by writing the alphabet in the correct order (Berninger et al., 1992, 1997; Graham and Weintraub, 1996; Medwell and Wray, 2014). However, these tasks can only be administered to children who have acquired writing skills (second grade or higher) and the rating of the above-mentioned categories is very subjective since there is no standard that would allow a comparison of the results between different age-groups. Furthermore, with the advent of new technologies researchers shifted to a more process-oriented approach to investigate handwriting (Rosenblum et al., 2003, 2006; Medwell and Wray, 2007; Tucha et al., 2008; Accardo et al., 2013; Gerth et al., 2016). These technologies provide an objective assessment of the dynamic subprocesses of handwriting (e.g., writing duration, in air time, writing velocity etc.; Marquardt and Mai, 1994; Tucha et al., 2008; Sumner et al., 2014; Gerth et al., 2016). Especially the number of inversions in velocity (NIVs) that describe the number of directional changes in velocity reflect how fluent and smooth handwriting movements are. Studies by Tucha et al. (2008; see also Tucha and Lange, 2005) have shown that directing attention to the writing movements increased the NIVs and hampered the automaticity of handwriting performance (even in adults). Thus, we believe that NIVs are an adequate and objective handwriting measure to quantify the level of automaticity in graphomotor execution and the amount of directed attention to the writing process.

Concering the comparison of the two writing surfaces—tablet and paper—a recent review article by Wollscheid et al. (2016) identified merely ten articles that compare the impact of writing tools (computer keyboards and tablet) vs. non-digital writing tools (pen and paper) on primary school students. The authors included studies that were published between 2005 and February 2015. Seven of the studies compared handwriting with typing. Only one article (Read et al., 2005) actually compared writing with a pen on a graphic tablet to using pencil and paper (and typing as a third condition). The 7 to 8 year old students wrote a story for about 12 min and were then given 2 min to edit their work. The stories were rated according to quality (teacher assessed) and quantity of writing (word count). However, this way of comparing the two media—tablet and paper—is quite product-oriented and cannot grasp the dynamics of graphomotor execution during writing on the two writing surfaces.

Only a few studies systematically investigated the question whether there is a difference between writing on a tablet and on paper. Alamargot and Morin (2015) studied second and ninth graders who wrote the alphabet and their own names on a tablet and on paper. Their results show that both groups wrote their names less legible and letter size was larger for both tasks on the tablet. The two groups were influenced differently by the two writing surfaces. The ninth graders showed faster writing speed and higher pen pressure whereas the second graders exhibited more pauses during writing on the tablet. A second study by Gerth et al. (2016) compared handwriting performance of adults on a tablet and on paper. Their findings reveal differences between writing on the two media that were partly modulated by the writing task. Even experienced writers, such as most adults, were influenced by the difference in friction between the writing surfaces. Interestingly, adults were able to adapt their graphomotor execution quickly to the smoother surface of the tablet by modulating their pen pressure and enlarging the writing size. Yet, there is no research that compared handwriting performance of participants without prior writing instruction (preschoolers) with that of beginning writers (second graders) and experienced writers (adults).

### The present study

The aim of the present study is to determine whether there are general and task-related effects of different levels of automaticity during writing on a tablet and on paper. To reach a comprehensive understanding of different levels of handwriting performance we chose the following three tasks with differing task demands assessing (1) graphomotor abilities—using continuous and repetitive patterns that participants had to copy, (2) visuomotor abilities—using a standardized test for which participants had to copy geometric forms and (3) automatic handwriting abilities—using a word-copying task. For all three tasks we evaluated handwriting quality (writing product) and handwriting dynamics (writing process). Measures of handwriting quality reflect influences of the writing surface on the handwriting performance, which are immediately visible to the writer. In contrast, the handwriting dynamics reflect subconscious motor and cognitive processes that can only be detected through handwriting measures recorded by the tablet. Further, we wanted to capture different levels of handwriting automaticity to investigate whether group differences could be due to a distinct adaptation to the smoother and unfamiliar writing surface (i.e., the tablet). Until now handwriting development research has focused on comparing adults' and children's handwriting performance. We added the group of preschoolers with very basic handwriting skills and conducted the study with three participant groups with different levels of handwriting automaticity (preschoolers, second graders, and adults). We expected that preschoolers perform worse regarding the handwriting quality and with lower automaticity in handwriting dynamics in all tasks compared to the second graders and adults. We predicted similar results for the second graders' handwriting performance compared to the one of the adults'. Taken together, we used a wide-ranging set of tasks to obtain a comprehensive picture on different dimensions of handwriting and to explore task-dependent adaptations to the writing surface. Handwriting quality and dynamics might be modulated by the participant's experience with writing on the tablet or paper and by the participant's level of handwriting automaticity.

## Methods and materials

### Participants

To capture the development in handwriting, we recruited three groups with varying levels of handwriting automaticity. Twenty-five preschoolers [17 female, mean age 5.4 years (*SD*: 0.6)] and 27 children in second grade [14 female, mean age 7.7 years (*SD*: 0.5)] were tested in this study. The preschoolers were recruited from three different kindergartens in Potsdam and the second graders from a day care center in Potsdam. The presented study also included a control group of 25 adults [21 female, mean age 21.8 years (*SD*: 2.6)] taken from an earlier study (Gerth et al., 2016). All participants were right-handed German native speakers and naïve to the purpose of the study. They had normal or corrected-to-normal vision. The parents of the children were informed about the study in an information letter and gave their written informed consent for the participation of their children. The study was approved by the ethics committee of the University of Potsdam (Reference number 41/2014) and it was conducted in accordance with the ethical standards laid down in the Declaration of Helsinki.

### Procedure

We conducted the study in two conditions: (1) writing with a Lenovo Pen on a ThinkPad X61 and (2) writing on a sheet of paper with an Intuous Inking Pen. To obtain the same handwriting measures as in condition (1) we placed the paper on a digitizer (Intuos4 XL DTP) and the digitizer was connected via an USB cable to a ThinkPad X61 (henceforth tablet). We could thus record performances with the same temporal and spatial resolution in both conditions. The digitizer and the tablet have tarnished plastic surfaces. The paper has a density of 80 g/m^2^. In order to level the height of the tablet with the forearm of the participant's writing hand we used a wooden frame (width: 62 cm, length: 46 cm, height: 3 cm). We set the sampling frequency of both devices to 133 Hz using the Wacom© software. The acquisition software was programmed in C# and XAML using Visual Studio Community 2013 Update 4 and the Windows Presentation Foundation runtime libraries provided by the Microsoft .NET Framework 4.5© Microsoft. In the methods study by Gerth et al. (2016) the friction of the two writing surfaces was quantified in an experimental set-up. The results showed that the friction of the paper surface was higher compared to the tablet surface (mean in writing velocity paper: 17.91 mm/s, tablet: 35.15 mm/s).

The preschoolers were tested individually in a quiet room in the kindergarten and the second graders in a silent room in the day care center. The adults' control group was tested in a silent laboratory at the University of Potsdam. All participants sat in a chair adjusted to their height in front of a table on which we positioned the tablet or paper on a digitizer. Half of the participants in each group started with condition (1), the other half with condition (2). Before the actual experiment, participants were familiarized with the medium by writing their first name and drawing circles around a dot. To prevent any bias from handedness the experimenter placed the pen in the middle of the tablet in front of each participant. Only right-handed participants were included in the study to prevent distorted results due to handedness. One session took approximately 20 min for all participants. The time between sessions varied between 2 and 19 days.

### Materials

We used three different tasks (used also by Gerth et al., 2016) measuring (a) graphomotor abilities, (b) visuomotor abilities, and (c) handwriting abilities (copying the phrase “Sonne und Wellen” [German for “sun and waves”]). Each task was performed twice by each participant, once on a tablet with a pen and in another session on paper attached to a tablet. We kept the writing space and the order of tasks parallel in both sessions.

#### Graphomotor abilities

**Figure 1:**
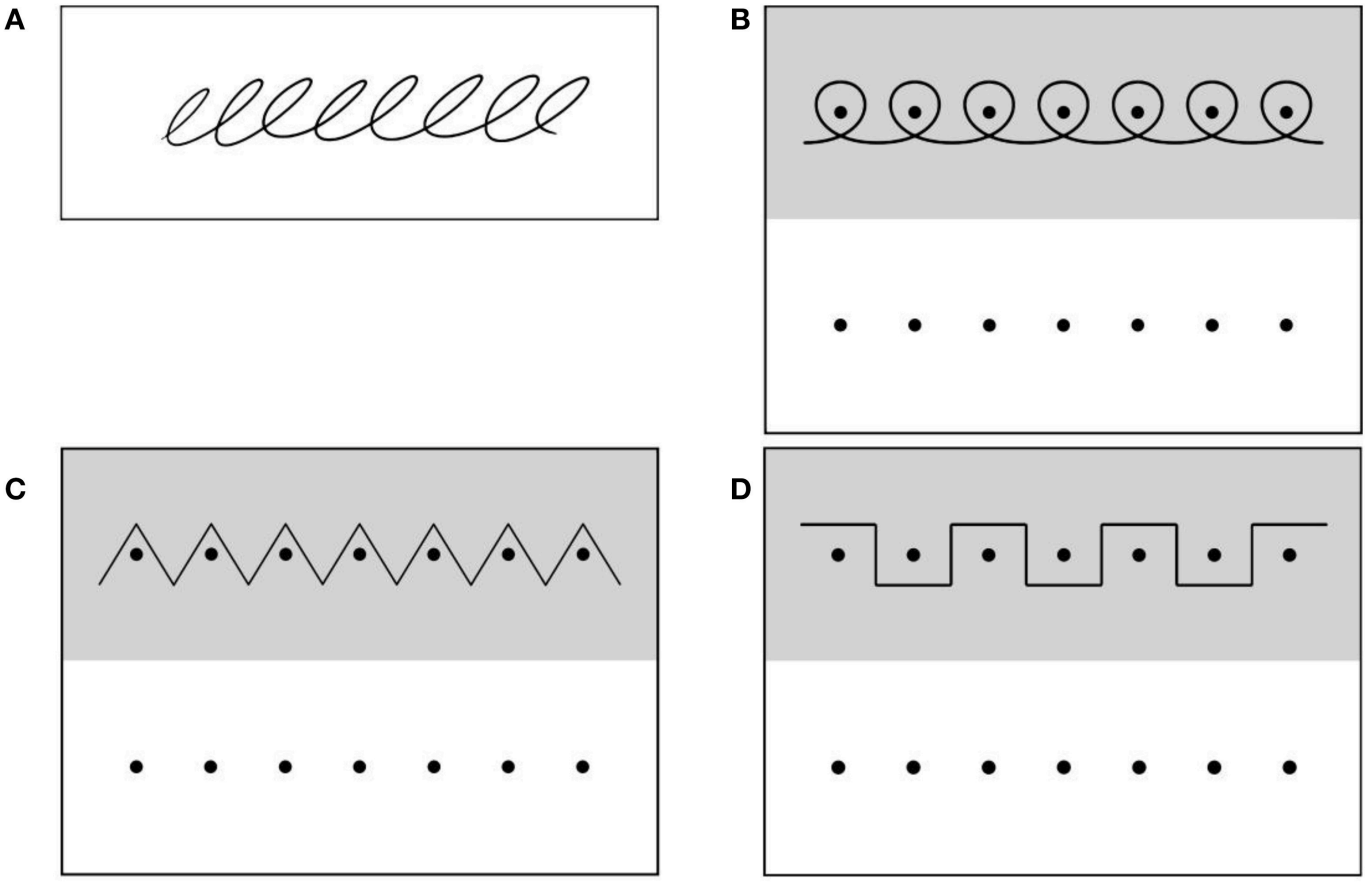
**Instruction (A): Copy the loop pattern on the next screen. (B–D)** Copy the pattern above in the space below. Reprinted from Gerth et al. (2016) with permission from Elsevier.

**Figure 2:**
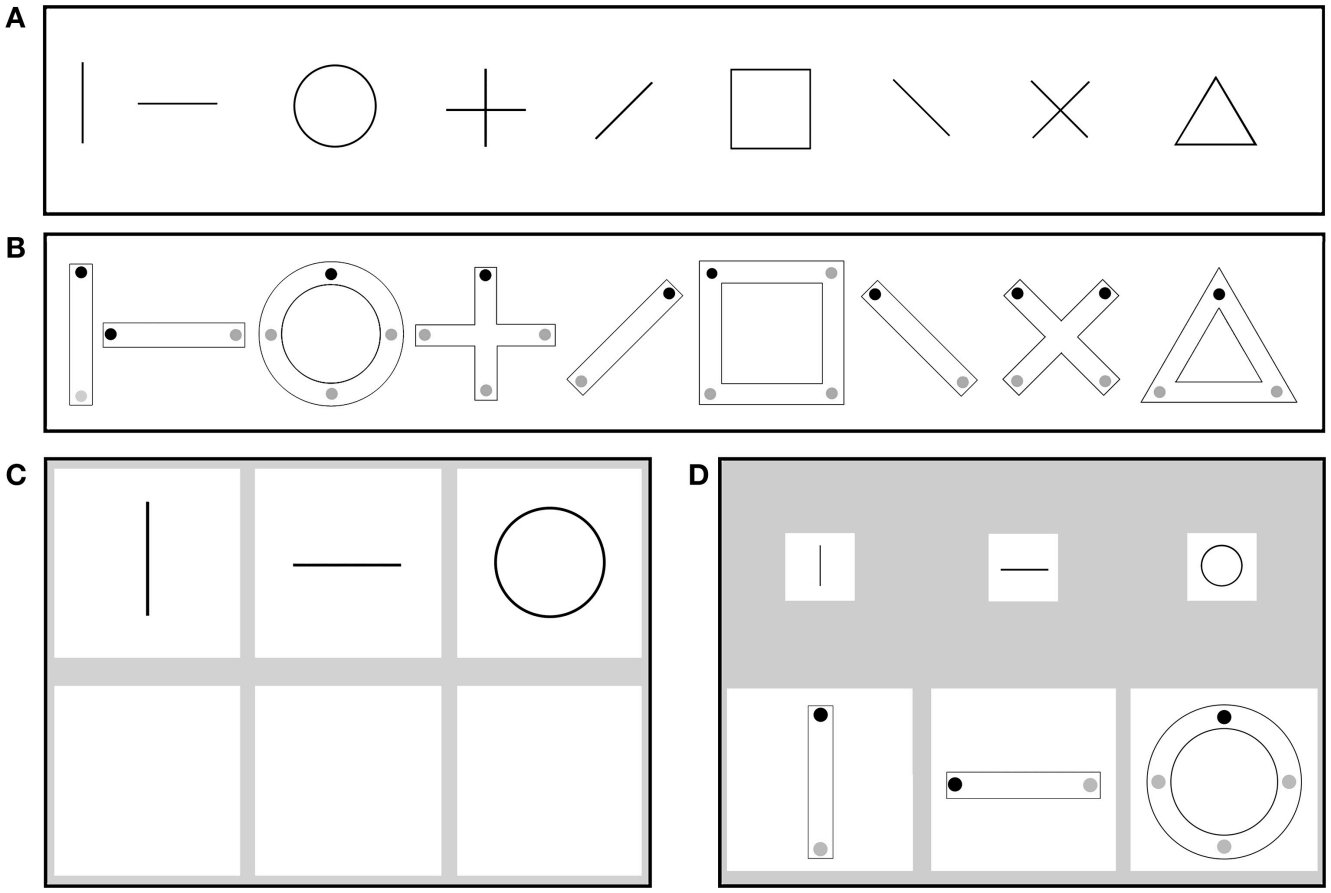
**The first nine forms of the Visual-Motor Integration task (A) and the Motor Coordination task (B) (Beery and Beery, 2010)**. The writing space of the VMI **(C)** and the MC **(D)**. Reprinted from Gerth et al. (2016) with permission from Elsevier.

**Figure 3:**
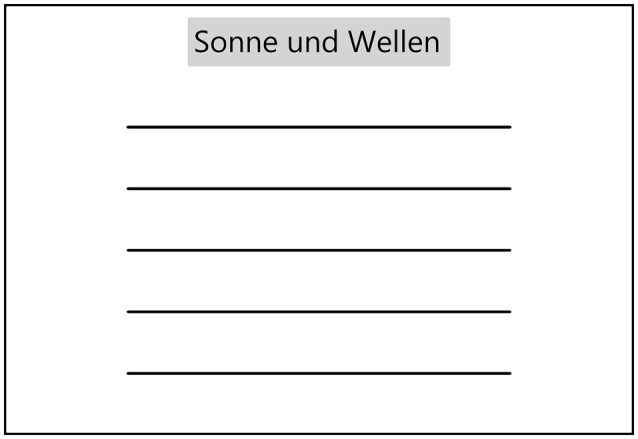
**The writing space for “Sonne und Wellen” (German, English “sun and waves”)**. Reprinted from Gerth et al. (2016) with permission from Elsevier.

**Figure 4:**
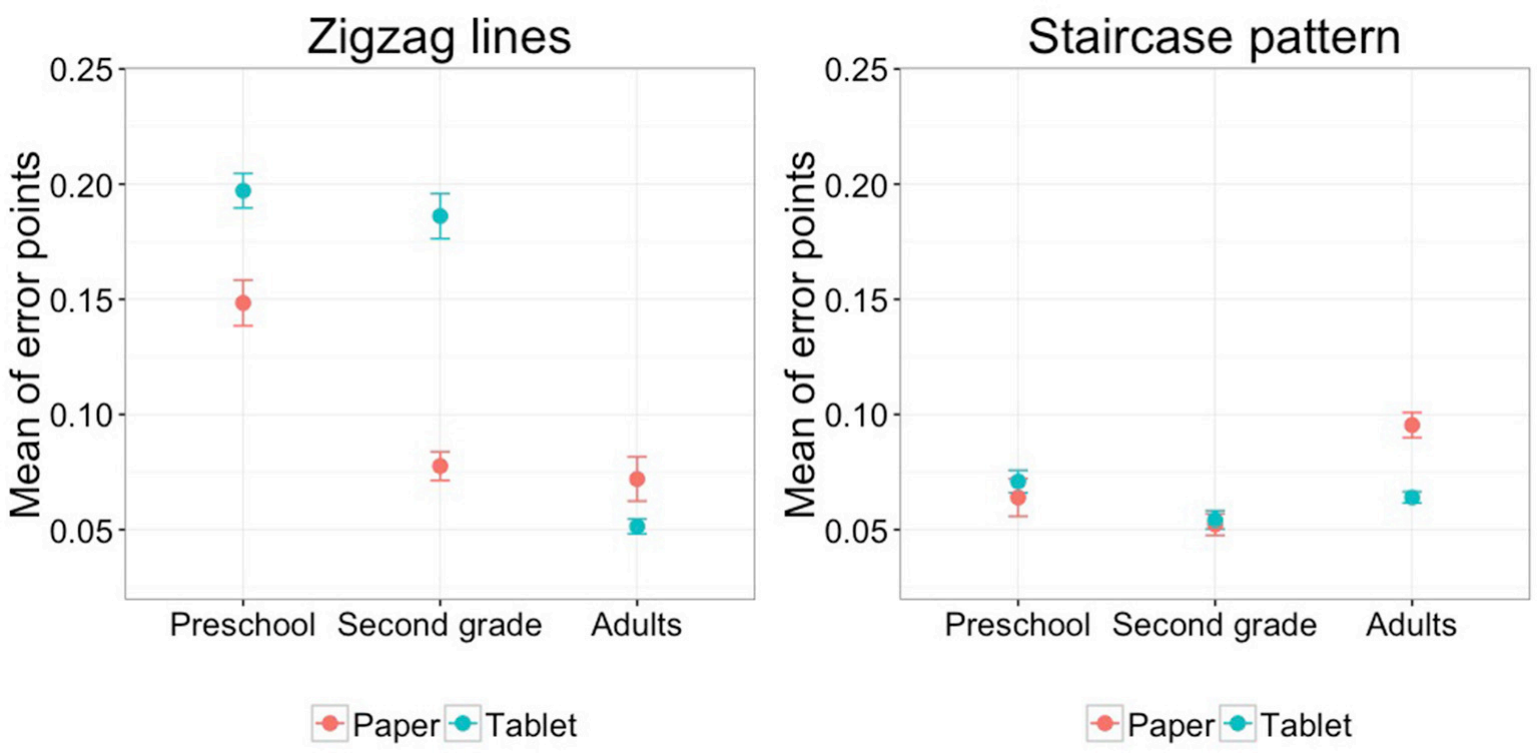
**The significant interaction between medium and group for the error points of zigzag lines and the staircase pattern (with standard errors)**.

In order to investigate graphomotor abilities we used four continuous and repetitive movement patterns: (1) loop patterns without constraints (Figure 1A), (2) loop patterns around dots (Figure 1B), (3) zigzag lines (Figure 1C), and (4) staircase patterns (Figure 1D). For the first task, the experimenter drew the loop pattern and the participant had to copy the movement on the next screen (Figure 1A). For all other tasks the pattern to copy was given in the upper half of the screen and the participant copied the pattern below. Each pattern was produced twice by the participant. The writing space for all four tasks had a size of 24.7 × 8.5 cm.

#### Visuomotor abilities

We were further interested in those visuomotor abilities that are known to predict handwriting measures. Therefore, we selected two tasks of the standardized test of visuomotor abilities, the Beery-Buktenica Developmental Test of Visual-Motor Integration (VMI) 6th Edition (Beery and Beery, 2010). This test is child-friendly and captures how visual perception and finger-hand movements are coordinated in children and adults, e.g., during handwriting (Volman et al., 2006). We used the first 9 items of the VMI and the Motor Coordination (MC) tasks because these forms can be mastered even by children who cannot write (Weil and Cunningham-Amundson, 1994). Since we tested preschool children without any prior instruction in writing this was an important criterion for item selection. The first 9 forms in both tasks (VMI and MC) are identical. We created a digital version of both tests to be able to track the handwriting process on a tablet.

For the first task, the VMI, participants had to copy geometric forms (Figure 2A) that were shown in the upper half of the screen (in groups of three items) into a square directly below (Figure 2C). Similarly, in the second task, the MC, participants traced a geometric form (Figure 2B) by connecting the dots (starting at the black dot) without crossing the double-lined path. The figures to be copied were presented in the upper half of the screen in a smaller scale which is in accordance with the guidelines of the standardized test (Figure 2D). Each square for both tasks had a size of 7.5 × 7.5 cm.

#### Handwriting abilities

Lastly, we investigated the process of writing the phrase “Sonne und Wellen” (German, in English “sun and waves”). This task was only administered with two participant groups, the second graders, and the adults, since the preschoolers had no prior writing instruction and could therefore not complete this writing task. The participants copied the phrase 10 times on given lines in their own handwriting speed. We did not constraint the type of handwriting—printed or cursive. The printed phrase was presented at the top of the screen to prevent any bias due to the participants' memory capacity (Figure 3). The lines were 15 cm long and the space between the lines was 2.4 cm (100 px).

#### Data analysis

To evaluate the handwriting product, we ran linear models on the error points (dependent variable) for each task with the factor Medium (tablet vs. paper) and the factor Group (preschool, second grade, adults) for the between-group comparisons using the software R version 3.0.1 (R Development Core Team, 2013).

For the analysis of the handwriting process measures, the *x*- and *y*-coordinates of the pen were recorded together with the time with a sampling frequency of 133 Hz. We smoothed the resulting velocity profiles by implementing the non-parametrical kernel estimation devised by Marquardt and Mai (1994) with the help of R-scripts (R version 3.0.1, R Development Team Core Team 2013). Then we computed the writing velocity by taking the first derivative of the *x*- and *y*-coordinates with respect to time. To obtain the number of inversions in velocity (NIVs) we calculated the sum of all NIVs per item. NIVs are sometimes calculated as the sum per up-stroke and down-stroke (Tucha et al., 2008). However, we changed this to the sum of all NIVs for one item because we did not exclusively test the writing of words.

We performed a standard outlier adjustment of the data based on the handwriting process measures by excluding data that were 3 standard deviations (*SD*'s) above the group mean for all handwriting measures (listed in Section Handwriting Process Measures). These data were mainly due to technical problems, misunderstandings of the instructions or other external factors. Additionally for writing velocity we excluded data 3 *SD*'s below the group mean. For the VMI and MC task the item complexity varied substantially, hence we excluded data based on the mean of the item instead of the group mean. In total we excluded 5.2% for the paper condition (preschool children: 6.6%, second graders: 5.9% and adults: 3.3%) and 5.2% for the tablet condition (preschool children: 4.8%, second graders: 6.1% and adults: 3.8%). In a next step we excluded a data point in the data set for the tablet condition if it had previously been removed for the paper condition and the other way around, because we were interested in the direct comparison of the two writing surfaces. Thereby we could apply repeated-measures for the statistical analyses without any problems due to missing data. In total we excluded 9.4% of the data.

We analyzed each task separately by applying linear mixed-effect models with repeated measures using the function lme() provided by the software R version 3.0.1 (R Development Core Team, 2013) and the nlme-package (Pinheiro et al., 2014). For each handwriting measure (dependent variable) we ran a separate model. The independent variables were the factor Medium (tablet vs. paper) and for the group-comparison the factor Group (preschool, second grade, adults). The models were fit using the maximum likelihood method (method = “ML”) and participants were used as random factors within the factor Medium (random = 1|~Participant/Medium). We log-transformed the writing duration and the in air time in order to avoid skewed distributions.

#### Handwriting product evaluation

To evaluate the quality of the produced items we scored the results of the tasks visually. The standardized tasks VMI and MC were evaluated according to the manual of the Beery-Buktenica Developmental Test of Visual-Motor Integration 6th Edition (Beery and Beery, 2010). Each geometric form was quantified by two raters (1—correctly copied item, 0—incorrectly copied item) and the total score quantifies how accurately the participants copied the forms.

For the graphomotor and the handwriting abilities task we created a scoring scheme that was inspired by the standardized Minnesota Handwriting Assessment, MHA (Reisman, 1999). We used 5 error categories for the graphomotor abilities and 4 error categories for the handwriting abilities. The 5 error categories for graphomotor abilities were: (1) pen lift during the task, (2) overlapping loops, (3) the lowest point of a loop is drawn lower than the highest point of any other loop (which means that the loops had to be drawn in a horizontal orientation), (4) a loop is unrecognizable, and (5) upside-down loops. To quantify the quality of the handwriting abilities task we created a similar rating scheme with 4 error categories: (1) legibility, (2) shape, (3) alignment in relation to the base line, and (4) spacing between letters. Each symbol (e.g., loop) and each letter (of “Sonne und Wellen”) was rated separately in each of the 5 error categories. For the handwriting task there are 14 letters in total for one item. The rater scored the legibility for each of the 14 letters and counted how often an error of legibility occurred. If a participant made 3 legibility errors, then 3 was divided by 14 (maximum of letters) to obtain the total error points score for legibility of this item (= 0.21). This scoring procedure was applied to all 4 error categories for the handwriting abilities. If there was no error in one of the categories the score was set to 0. In the end the scores of the error categories were summed up and divided by 4 (= total number of error categories for handwriting abilities) to obtain the total error score for an item (with two decimal places). We used the same scoring scheme for loops without dots (maximum number of loops was equal to the number of loops drawn), loops with dots (maximum number of loops: 7), zigzag lines (maximum number of triangles: 7), and the staircase pattern (maximum number was set to number of possible strokes: 13).

#### Handwriting process measures

To evaluate the handwriting process we calculated the following handwriting measures.

*Writing duration:* the time in milliseconds (ms) that the pen is on the surface of the tablet or paper (pressure > 0). This gives an indication of temporal performance and is linked to average velocity (Rosenblum et al., 2003).

*Writing velocity*: in millimeter per second (mm/s). This measure is used to evaluate the fluidity in handwriting performance (Rosenblum et al., 2003).

*In air time*: the time in ms that the pen is above the surface (distance < 1 cm). This measure indicates breaks in writing and might be linked to higher level processes (Rosenblum et al., 2003; Sumner et al., 2013).

*Number of strokes:* determines continuous movements until the pen is lifted from the surface (pressure = 0). A large number of strokes might reveal irregular and non-automatized writing (Tucha et al., 2008).

*Number of inversions in velocity (NIV):* indicate the degree of handwriting automaticity and are related to the number of accelerations and decelerations during writing. While low NIVs characterize an automatized and smooth movement, higher NIVs are associated with a lesser degree of automaticity, for instance when adults are asked to mentally track their own handwriting movements (Marquardt et al., 1996; Tucha et al., 2008).

## Results

At first we will present the results of our visual evaluation of the quality of the produced items (Section Handwriting Product Evaluation) and then examine the results of the handwriting process measures (Section Handwriting Process Measures). For both parts we will firstly review the results of the comparison between the two surfaces (tablet vs. paper) to show differences in graphomotor execution between the media and secondly the results for the between-group analyses to investigate differences in the level of handwriting acquisition.

### Handwriting product evaluation

#### Graphomotor abilities

Table 1 presents a summary of the data and statistical effects for our scoring of the handwriting products for each of the tasks. Regarding the graphomotor abilities we found differences between the execution on the tablet and paper for loops with dots only for the preschool children (*p* = 0.048) such that they obtained more error points on the tablet compared to paper. For zigzag lines all groups showed differences between the two writing surfaces (preschool: *p* < 0.001; second grade: *p* < 0.001; adults: *p* = 0.039), the preschoolers and second graders received more error points in the tablet condition while the adults showed the opposite pattern with more error points for the paper condition. For the last task, the staircase pattern, only the adults showed a significant difference between the media (*p* < 0.001) with more error points when executing the task on paper compared to on the tablet.

**Table 1:** **Means and standard deviations in parentheses for the scoring of the handwriting product**.

	**Preschool**	**Second grade**	**Adults**
**LOOPS WITHOUT DOTS—ERROR POINTS**			
Paper	0.175 (0.134)	0.072 (0.112)	0.042 (0.017)
Tablet	0.186 (0.114)	0.077 (0.038)	0.039 (0.018)
*p*-value	0.695	0.756	0.498
*b*-value	0.010	0.005	−0.002
**LOOPS WITH DOTS—ERROR POINTS**			
Paper	0.132 (0.099)	0.061 (0.032)	0.034 (0.012)
Tablet	0.204 (0.232)	0.078 (0.059)	0.038 (0.014)
*p*-value	0.048^*^	0.066	0.079
*b*-value	0.072	0.017	0.005
**ZIGZAG LINES—ERROR POINTS**			
Paper	0.148 (0.070)	0.078 (0.046)	0.072 (0.068)
Tablet	0.197 (0.051)	0.186 (0.072)	0.051 (0.023)
*p*-value	<0.001^*^	<0.001^*^	0.044^*^
*b*-value	0.049	0.109	−0.021
**STAIRCASE PATTERN—ERROR POINTS**			
Paper	0.064 (0.054)	0.052 (0.034)	0.095 (0.039)
Tablet	0.071 (0.033)	0.054 (0.029)	0.064 (0.017)
*p*-value	0.463	0.741	<0.001^*^
*b*-value	0.007	0.002	−0.031
**VISUAL MOTOR INTEGRATION (VMI)—ACCURACY IN %**			
Paper	92.9 (25.8)	99.6 (6.2)	100 (0)
Tablet	85.0 (35.7)	96.5 (18.3)	99.6 (6.7)
*p*-value	<0.001^*^	0.011^*^	0.318
*b*-value	−0.078	−0.031	−0.004
**MOTOR COORDINATION (MC)—ACCURACY IN %**			
Paper	94.7 (22.5)	98.9 (10.7)	100 (0)
Tablet	85.5 (35.3)	91.6 (27.8)	98.7 (11.5)
*p*-value	<0.001^*^	<0.001^*^	0.083
*b*-value	−0.092	−0.073	−0.013
**WRITING “SUN AND WAVES”—ERROR POINTS**			
Paper	–	0.116 (0.094)	0.125 (0.109)
Tablet		0.125 (0.085)	0.165 (0.122)
*p*-value		0.220	<0.001^*^
*b*-value		0.010	0.040

*The p-value refers to the comparison for Medium (tablet vs. paper). The b-value refers to the regression coefficient of the tablet condition in comparison to paper. The asterisk indicates significant effects below an alpha-level of 0.05*.

The results of between-group analyses show that preschoolers obtained more error points compared to adults for all four graphomotor ability tasks (all *p* < 0.001; loops without dots *b* = −0.038, loops with dots *b* = −0.067, zigzag lines *b* = −0.069, staircase pattern *b* = −0.038) and preschoolers produced more error points than second graders for loops without dots (*p* < 0.001, *b* = 0.104), loops with dots (*p* < 0.001, *b* = −0.071), and zigzag lines (*p* < 0.001, *b* = −0.071). The comparison of second graders and adults yielded a significantly worse performance for second graders only for the staircase pattern (*p* < 0.001, *b* = 0.043). Additionally we obtained significant interactions between the factor Medium (tablet vs. paper) and Group (preschool, second grade, adults) for three of the tasks: (1) for loops with dots between preschoolers and adults (*p* = 0.022, *b* = −0.067; preschoolers showed a difference in error points between paper and tablet while there was no such difference for the adults), (2) for zigzag lines between preschoolers and adults (*p* < 0.001, *b* = −0.069) as well as second graders and adults (*p* < 0.001, *b* = −0.129; adults exhibited more error points on paper but preschoolers and second graders produced more error points on the tablet) and between preschoolers and second graders (*p* < 0.001, *b* = 0.060; second graders obtained a larger increase in error points between tablet and paper than preschoolers), and (3) for the staircase pattern between preschoolers and adults (*p* < 0.001, *b* = −0.383) as well as second graders and adults (*p* < 0.001, *b* = −0.033; adults showed a significant difference in error points between tablet and paper whereas preschoolers and second graders did not). Figure 4 visualizes the significant interactions (2) and (3).

#### Visuomotor abilities

Two raters evaluated the visuomotor ability tasks, VMI and MC, according to the test manual (Beery and Beery, 2010). The accuracy data describes how accurately the participants copied the geometric forms (VMI) or traced the geometric forms without crossing the double-lined path (MC). The comparison between tablet and paper yielded differences only for the children groups for the VMI (preschool: *p* < 0.001; second grade: *p* = 0.011) as well as the MC (preschool: *p* < 0.001; second grade: *p* < 0.001) such that both groups showed a better performance (fewer error points) on paper compared to the tablet condition for both tasks. The adults were at ceiling performance and exhibited no differences between the two media.

The between-group analyses revealed differences in group performances for both tasks (VMI and MC) between preschoolers and adults (VMI: *p* < 0.001, *b* = −0.074; MC: *p* = 0.009, *b* = 0.053) as well as between preschoolers and second graders (VMI: *p* < 0.001, *b* = −0.067; MC: *p* = 0.033, *b* = −0.042) mirroring the fact that preschoolers performed less accurate than second graders and adults; additionally second graders were less accurate than adults in both tasks. Furthermore, we found significant interactions between Medium (tablet vs. paper) and Group (preschool, second grade, adults) for both tasks. For VMI the interaction was significant between preschoolers and adults (*p* = 0.005, *b* = −0.074) meaning that we did not find a difference in accuracy between-media for adults but for preschoolers. For MC the analyses revealed two interactions: one between preschoolers and adults (*p* = 0.006, *b* = 0.079) and a second one between second graders and adults (*p* = 0.033, *b* = 0.059) showing that both children groups exhibited significant differences in performance on the tablet and on paper whereas the adults showed no such between-media difference in accuracy.

#### Handwriting abilities

The results of writing the phrase “Sonne und Wellen” showed differences between writing on paper vs. on the tablet only for the adults' group (*p* < 0.001) who wrote less well on the tablet than on paper. The performance of the second graders was not significantly different between the media (*p* = 0.220) but pointed into the same direction as the results of adults.

When comparing the second graders' performance to that of the adults we found only a significant interaction between Medium (tablet vs. paper) and Group (second grade, adults; *p* = 0.016, *b* = −0.031) mirroring the fact that adults showed a difference in error points between tablet and paper whereas the second graders exhibited no such difference.

### Handwriting process measures

#### Graphomotor abilities

Table 2 presents the descriptive data and statistical effects for the writing measures of the graphomotor abilities for the three groups. We will review the results in the order of the writing measures.

**Table 2:** **Means and standard deviations in parentheses for writing measures of the graphomotor abilities**.

	**Writing duration (ms)**	**In air time (ms)**	**Number of pen lifts**	**Velocity (mm/s)**	**NIVs**
**LOOPS WITHOUT DOTS**					
**Preschool**					
Paper	11918.97 (5832.35)	394.29 (1054.81)	1.69 (1.32)	41.40 (20.29)	76.94 (41.51)
Tablet	8741.83 (3988.76)	37.20 (181.56)	1.06 (0.24)	54.78 (22.61)	52.46 (26.18)
*p*-value	0.012^*^	0.039^*^	0.038^*^	0.026^*^	0.005^*^
*b*-value	−0.305	−1.789	−0.762	12.264	−24.762
**Second grade**					
Paper	9548.36 (5591.74)	127.50 (358.57)	1.36 (0.79)	59.21 (25.31)	60.43 (34.95)
Tablet	6696.05 (2497.08)	128.81 (307.44)	1.19 (0.40)	80.49 (20.93)	40.64 (14.84)
*p*-value	0.077	0.638	0.524	0.014^*^	0.024^*^
*b*-value	−0.239	0.370	−0.104	17.825	−18.875
**Adults**					
Paper	4760.90 (1273.86)	0	1	104.55 (36.75)	34.88 (7.46)
Tablet	4549.95 (934.84)	0	1	125.06 (31.97)	32.30 (5.75)
*p*-value	0.216	−	−	<0.001^*^	0.021^*^
*b*-value	−0.047	−	−	23.979	−2.920
**LOOPS WITH DOTS**					
**Preschool**					
Paper	15487.32 (5731.07)	355.86 (1012.18)	1.43 (1.04)	28.64 (10.78)	95.84 (37.34)
Tablet	13698.38 (4273.72)	314.38 (570.60)	1.49 (0.80)	37.24 (11.94)	78.68 (25.40)
*p*-value	0.226	0.226	0.909	0.011^*^	0.039^*^
*b*-value	−0.108	1.066	0.025	8.130	−17.300
**Second grade**					
Paper	12883.36 (4278.60)	157.79 (302.76)	1.30 (0.55)	31.47 (13.97)	75.49 (28.41)
Tablet	11376.30 (3010.24)	276.87 (620.24)	1.38 (0.74)	41.80 (14.93)	63.91 (19.20)
*p*-value	0.173	0.980	0.499	0.001^*^	0.125
*b*-value	−0.091	−0.022	0.096	9.984	−10.038
**Adults**					
Paper	8168.95 (2420.36)	0	1	43.03 (16.07)	43.36 (12.50)
Tablet	7636.17 (2165.83)	0	1	53.17 (18.17)	42.63 (9.90)
*p*-value	0.026^*^	−	−	<0.001^*^	0.249
*b*-value	−0.099	−	−	11.853	−2.960
**ZIGZAG LINES**					
**Preschool**					
Paper	11609.78 (3801.26)	393.22 (1007.98)	1.68 (1.66)	34.13 (15.01)	67.46 (27.90)
Tablet	9993.20 (2860.59)	46.78 (184.47)	1.07 (0.26)	48.89 (17.22)	52.20 (16.32)
*p*-value	0.025^*^	0.105	0.056	<0.001^*^	0.005^*^
*b*-value	−0.122	−1.337	−0.696	14.413	−13.913
**Second grade**					
Paper	10986.82 (4088.58)	85.69 (174.71)	1.18 (0.49)	33.37 (14.80)	60.47 (25.23)
Tablet	9818.04 (2784.52)	232.79 (306.19)	1.31 (0.60)	47.81 (19.03)	51.09 (14.44)
*p*−value	0.039^*^	0.212	0.558	<0.001^*^	0.015^*^
*b*−value	−0.104	0.995	0.083	14.745	−9.917
**Adults**					
Paper	7938.73 (2511.17)	0	1	40.54 (15.37)	42.52 (12.70)
Tablet	7552.21 (2467.75)	0	1	51.83 (18.95)	40.50 (10.45)
*p*-value	0.188	−	−	<0.001^*^	0.211
*b*-value	−0.063	−	−	12.636	−2.348
**STAIRCASE PATTERN**					
**Preschool**					
Paper	12785.67 (4419.22)	264.90 (894.64)	1.43 (1.40)	16.84 (6.37)	76.64 (29.77)
Tablet	14368.05 (6587.95)	357.93 (1171.46)	1.36 (0.91)	20.29 (8.83)	78.88 (29.73)
*p*-value	0.263	0.816	0.509	0.114	0.789
*b*-value	0.094	0.180	−0.250	2.870	1.896
**Second grade**					
Paper	12917.60 (4562.05)	142.18 (373.93)	1.20 (0.40)	13.55 (5.45)	78.86 (29.11)
Tablet	11619.42 (3897.41)	323.88 (608.87)	1.46 (0.81)	18.62 (7.33)	70.20 (24.67)
*p*-value	0.200	0.056	0.033^*^	<0.001^*^	0.214
*b*-value	−0.085	1.553	0.352	4.715	−6.926
**Adults**					
Paper	8301.65 (2647.80)	48.86 (288.61)	1.10 (0.57)	20.76 (8.14)	48.88 (18.13)
Tablet	7663.69 (2462.25)	0	1	26.78 (10.32)	44.43 (15.60)
*p*-value	0.051	−	−	<0.001^*^	0.047^*^
*b*-value	−0.090	−	−	6.610	−5.484

*The p-value refers to the comparison for Medium (tablet vs. paper). The b-value refers to the regression coefficient of the tablet condition in comparison to paper. The asterisk indicates significant effects below an alpha-level of 0.05*.

The writing duration was longer on paper than on tablet for loops without dots for the preschool children (*p* = 0.012), for loops with dots for the adults (*p* = 0.026), and for zigzag lines for the two children groups (preschool: *p* = 0.025; second grade: *p* = 0.039). Regarding in air time we found longer in air times for the paper condition than in the tablet condition for loops without dots for the preschool children (*p* = 0.039)^1^. Similarly we found more pen lifts for the paper condition compared to the tablet condition for loops without dots for the preschool children (*p* = 0.038) and for the staircase pattern the second graders lifted the pen more often in the tablet condition compared to paper (*p* = 0.033). The writing velocity was higher on the tablet than on paper for all tasks and all groups (all *p* < 0.026) except for the staircase pattern in the preschool children (*p* = 0.114). There were significantly fewer NIVs in the tablet condition compared to paper for loops without dots in all groups (preschool: *p* = 0.005; second grade: *p* = 0.024; adults: *p* = 0.021), for loops with dots only in the preschool group (*p* = 0.039), for zigzag lines in both children groups (preschool: *p* = 0.005; second grade: *p* = 0.015), and for the staircase pattern only in the adults group (*p* = 0.047).

Earlier research found that a smoother handwriting movement (= higher velocity) is associated with fewer NIVs (= more automatized and smoother movement; Meulenbroek and Van Galen, 1990; Gerth et al., 2016), therefore we computed Kendall's tau correlations^2^ between writing velocity and NIVs. For all four tasks and all three groups these two handwriting measures were negatively correlated, meaning that a smoother movement produced fewer NIVs (all *p* < 0.001; loops without dots: preschool τ = −0.66, second grade τ = −0.58, adults τ = −0.33; loops with dots: preschool τ = −0.67; second grade τ = −0.63; adults τ = −0.62; zigzag lines: preschool τ = −0.53; second grade τ = −0.57; adults τ = −0.59; staircase pattern: preschool τ = −0.71; second grade τ = −0.61; adults τ = −0.68).

**Figure 5:**
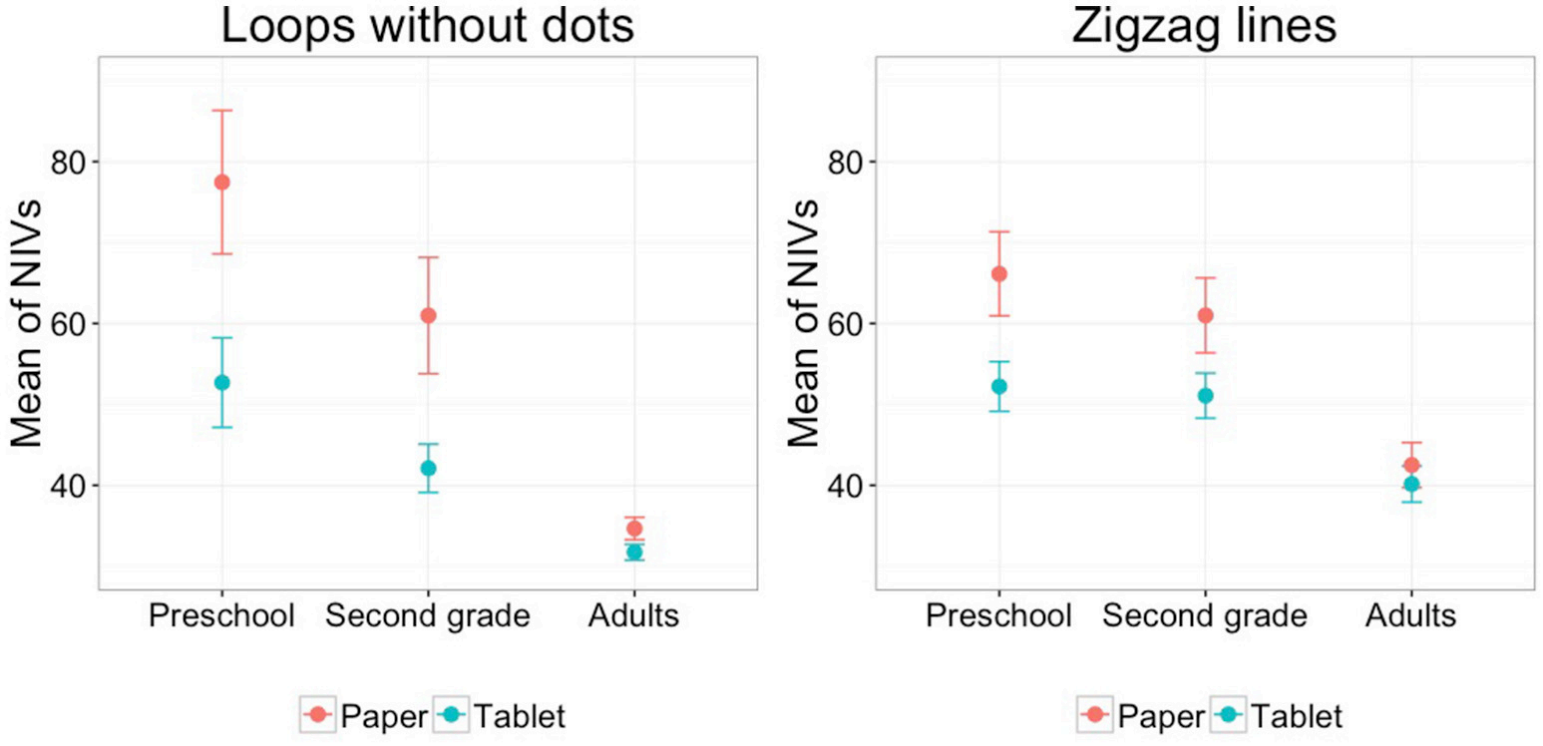
**The significant interaction between medium and group for the NIVs of loops without dots and zigzag lines (with standard errors)**.

Results of the between-group analyses for writing duration revealed for all four tasks that preschoolers wrote longer than adults (all *p* < 0.001, loops without dots *b* = −0.846, loops with dots *b* = −0.600, zigzag lines *b* = −0.373, staircase pattern *b* = −0.463) and preschoolers wrote longer than second graders for loops without dots (*p* = 0.034, *b* = −0.252) and loops with dots (*p* = 0.033, *b* = −0.193) as well as second graders wrote longer than adults for all four tasks (all *p* < 0.001, loops without dots *b* = −0.594, loops with dots *b* = −0.408, zigzag lines *b* = −0.331, staircase pattern *b* = −0.431). For in air time we found that for loops without dots, loops with dots and zigzag lines preschoolers lifted the pen longer than adults (all *p* < 0.008, loops without dots *b* = −2.351, loops with dots *b* = −2.002, zigzag lines *b* = −2.077) and second graders produced longer in air times than adults for loops without dots (*p* = 0.011, *b* = −1.552) and loops with dots (*p* = 0.003, *b* = −2.439). We also found significant interactions for in air time between Medium (tablet vs. paper) and Group (preschool, second grade, adults) for loops without dots between preschoolers and adults (*p* = 0.036, *b* = 1.789; preschoolers show a significant difference between tablet and paper while there was no difference for adults) and for zigzag lines between preschoolers and second graders [*p* = 0.007, *b* = 2.333; preschoolers exhibited numerically longer in air times for paper whereas second graders produced (numerically) longer in air times in the tablet condition]. For number of pen lifts we found that preschoolers lifted the pen more often than adults for all four tasks (all *p* < 0.05, loops without dots *b* = −0.833, loops with dots *b* = −0.525, zigzag lines *b* = −0.761, staircase pattern *b* = −0.574), preschoolers produced more pen lifts than second graders for loops without dots (*p* = 0.019, *b* = −0.500) and for zigzag lines (*p* = 0.015, *b* = −0.511). Additionally, we found significant interactions for loops without dots between preschoolers and adults (*p* = 0.008, *b* = 0.762; preschoolers showed a difference in pen lifts between the two writing surface whereas the adults did not lift the pen for either of the two) and between preschoolers and second graders (*p* = 0.027, *b* = 0.658; preschoolers show a significant difference in the number of pen lifts between tablet and paper whereas the second graders showed no difference), as well as for zigzag lines between preschoolers and adults (*p* = 0.026, *b* = 0.696; preschoolers lifted the pen numerically more often for paper whereas the adults showed no difference between media) and between preschoolers and second graders (*p* = 0.010, *b* = 0.779; preschoolers lifted the pen numerically more often for paper whereas second graders produced more pen lifts for the tablet). For writing velocity we found that preschoolers wrote significantly slower than adults for loops without dots (*p* < 0.001, *b* = 59.970) and loops with dots (*p* < 0.001, *b* = 12.235), further preschoolers wrote slower than second graders for loops without dots (*p* = 0.010, *b* = 18.546), and second graders wrote slower than adults for loops without dots (*p* < 0.001, *b* = 41.424), loops with dots (*p* = 0.022, *b* = 9.260), and the staircase pattern (*p* = 0.002, *b* = 6.964). For the NIVs we found that preschoolers produced significantly more NIVs than adults for all tasks (*p* < 0.001, loops without dots *b* = −42.792, loops with dots *b* = −52.080, zigzag lines *b* = −23.630, staircase pattern *b* = −31.089); preschoolers also produced more NIVs than second graders for loops without dots (*p* = 0.026, *b* = −16.473) and loops with dots (*p* = 0.002, *b* = −21.946) and second graders produced more NIVs than adults for all four tasks (all *p* < 0.001, loops without dots *b* = −26.319, loops with dots *b* = −30.134, zigzag lines *p* = −18.500, staircase pattern *b* = −28.689). Furthermore, we found significant interactions for Medium and Group between preschoolers and adults for loops without dots (*p* = 0.018, *b* = 21.842; preschoolers showed a bigger difference between the writing surfaces than the adults and performed worse than adults) and zigzag lines (*p* = 0.012, *b* = 11.565; preschoolers exhibited a difference in NIVs between tablet and paper whereas the adults showed no such difference). We illustrate these interactions between Medium and Group in Figure 5.

#### Visuomotor abilities

Table 3 shows a summary of the data and statistical effects for the handwriting measures of the VMI and MC tests. Only the adults wrote significantly longer on the computer compared to paper for the MC (*p* = 0.028). The writing velocity was higher on the tablet for all groups for the VMI (preschool: *p* < 0.001; second grade: *p* = 0.002; adults: *p* < 0.001). For the MC we found a higher writing velocity on the tablet only for the children groups (preschool: *p* < 0.001; second grade: *p* = 0.037). Regarding the NIVs only the preschool children produced more NIVs on paper compared to the tablet (*p* = 0.030).

**Table 3:** **Means and standard deviations in parentheses for writing measures of the visuomotor abilities**.

	**Writing duration (ms)**	**In air time (ms)**	**Number of pen lifts**	**Velocity (mm/s)**	**NIVs**
**VISUAL MOTOR INTEGRATION (VMI)**					
**Preschool**					
Paper	1728.87 (1210.87)	119.44 (244.08)	1.31 (0.54)	33.85 (19.08)	11.81 (7.98)
Tablet	1596.60 (1190.47)	172.12 (420.25)	1.31 (0.57)	42.86 (26.10)	10.11 (7.20)
*p*-value	0.157	0.960	1	<0.001^*^	0.030^*^
*b*-value	−0.095	0.014	0.000	9.008	−1.702
**Second grade**					
Paper	1935.90 (1380.33)	120.38 (243.92)	1.27 (0.48)	31.06 (21.60)	12.78 (9.51)
Tablet	1706.09 (1302.76)	161.63 (322.23)	1.31 (0.56)	42.02 (27.26)	10.83 (8.22)
*p*-value	0.099	0.564	0.409	0.002^*^	0.061
*b*-value	−0.140	0.153	0.042	6.976	−1.939
**Adults**					
Paper	1830.15 (1243.23)	219.59 (400.72)	1.45 (0.80)	29.88 (19.19)	11.79 (7.80)
Tablet	1792.31 (1300.92)	200.85 (359.22)	1.35 (0.62)	37.04 (23.89)	10.51 (7.78)
*p*-value	0.562	0.586	0.104	<0.001^*^	0.107
*b*-value	−0.033	−0.160	−0.115	6.976	−1.135
**MOTOR COORDINATION (MC)**					
**Preschool**					
Paper	3971.81 (2179.46)	394.59 (688.39)	1.50 (0.79)	15.04 (7.13)	27.87 (16.39)
Tablet	3819.11 (2416.13)	376.71 (778.39)	1.44 (0.78)	18.71 (8.69)	25.13 (16.19)
*p*-value	0.222	0.291	0.520	<0.001^*^	0.105
*b*-value	−0.076	−0.351	−0.051	3.674	−2.742
**Second grade**					
Paper	3995.59 (2470.78)	312.07 (530.39)	1.48 (0.75)	15.17 (7.40)	27.86 (17.27)
Tablet	4040.81 (2462.22)	308.78 (558.84)	1.42 (0.70)	17.19 (7.96)	25.85 (15.69)
*p*-value	0.866	0.572	0.388	0.037^*^	0.200
*b*-value	0.010	−0.175	−0.061	1.947	−2.014
**Adults**					
Paper	2582.54 (1546.54)	199.66 (380.76)	1.40 (0.73)	21.53 (11.40)	16.77 (9.98)
Tablet	2938.22 (1694.12)	232.25 (429.96)	1.41 (0.71)	21.00 (10.29)	17.02 (10.55)
*p*-value	0.028^*^	0.682	0.946	0.964	0.969
*b*-value	0.127	0.119	−0.005	−0.044	−0.038

*The p-value refers to the comparison for Medium (tablet vs. paper). The b-value refers to the regression coefficient of the tablet condition in comparison to paper. The asterisk indicates significant effects below an alpha-level of 0.05*.

The correlation analyses between writing velocity and NIVs revealed an inverse relationship between these two measures (all *p* < 0.001; VMI: preschool τ = –0.37, second grade τ = −0.39, adults τ = −0.40; MC: preschool τ = –0.38, second grade τ = –0.39, adults τ = –0.34).

The between-group analyses of writing duration revealed for the MC that preschoolers wrote longer than adults (*p* < 0.001, *b* = 0.445) and longer than second graders (*p* < 0.001, *b* = 0.425). Further the interaction between Medium (tablet vs. paper) and Group was significant for the MC between preschoolers and adults (*p* = 0.016, *b* = –0.204; preschoolers show no difference between the two writing surfaces whereas adults wrote longer on the tablet than paper). There were no significant effects for in air time. For the number of pen lifts we found for the VMI that preschoolers lifted the pen less often than adults (*p* = 0.003, *b* = 0.134) and more often than the second graders (*p* = 0.003, *b* = −0.177). For writing velocity we found for the MC that preschoolers and second graders wrote faster than adults (both *p* < 0.001, preschoolers vs. adults *b* = −6.212, second graders vs. adults *b* = −6.126). Further we found an interaction for the MC between preschoolers and adults (*p* = 0.002, *b* = 3.720; preschoolers wrote faster on the tablet whereas adults show no difference between the writing surfaces). For NIVs we found for the MC that preschoolers and second graders produced more NIVs than adults (both *p* < 0.001, preschoolers vs. adults *b* = 11.160, second graders vs. adults *b* = 11.242).

Both tasks contained the same set of items, therefore we conducted additional analyses with the factor Task (VMI vs. MC) to check directly for task differences in graphomotor demands. We found a main effect for Medium (tablet vs. paper) for writing velocity in all groups (preschool: *p* < 0.001, *b* = 6.361; second grade: *p* = 0.003, *b* = 6.279; adults: *p* < 0.002, *b* = 3.482) and for NIVs for preschool children (*p* = 0.020, *b* = −2.222). The factor Task yielded significant differences between VMI and MC for all writing measures for the preschoolers (writing duration: *p* < 0.001, *b* = 0.908, pause duration: *p* = 0.001, *b* = 0.695, number of pen lifts: *p* = 0.001, *b* = 0.155, velocity: *p* < 0.001, *b* = −21.534, NIVs: *p* < 0.001, *b* = 16.077) and second graders (writing duration: *p* < 0.001, *b* = 0.852, pause duration: *p* = 0.002, *b* = 0.635, number of pen lifts: *p* < 0.001, *b* = 0.155, velocity: *p* < 0.001, *b* = −20.415, NIVs: *p* < 0.001, *b* = 15.005) and for writing duration (*p* < 0.001, *b* = 0.453), velocity (*p* < = 0.001, *b* = −10.643) and NIVs (*p* < 0.001, *b* = 5.465) in the adult group. For the MC (compared to the VMI) participants wrote longer and slower and produced more NIVs. Additionally, the children groups lifted the pen for a longer time and more often for the MC than the VMI. The interaction between Medium and Task was significant for writing velocity for all groups (*p* < 0.001, preschoolers *b* = −9.076, second graders *b* = −9.077, adults *b* = −7.060) such that participants wrote faster on the tablet than on paper for the VMI, but there was no difference for the MC for the adult group and a smaller difference for velocity between-media for the children groups.

#### Handwriting abilities

Table 4 presents a summary of the data and statistical effects for the handwriting measures of writing the phrase “Sonne und Wellen.” We administered this task only to the second graders and adults because the preschoolers were not capable of writing words. We found longer writing durations on the tablet compared to paper for both groups (both *p* < 0.001). Only adults showed a longer in air time on the tablet (*p* = 0.002) and more pen lifts on paper (*p* = 0.031). Both groups exhibited a higher velocity on the tablet compared to paper (both *p* < 0.001). For the NIVs only adults produced more NIVs on the tablet than on paper (*p* = 0.010).

**Table 4:** **Means and standard deviations in parentheses for writing measures of writing the phrase “sun and waves”**.

	**Writing duration (ms)**	**In air time (ms)**	**Number of pen lifts**	**Velocity (mm/s)**	**NIVs**
**WRITING “SUN AND WAVES”**					
**Second grade**					
Paper	14753.40 (3446.42)	2664.79 (1232.79)	5.26 (1.33)	14.16 (4.91)	117.24 (17.27)
Tablet	17068.82 (4003.57)	3076.98 (1518.31)	5.38 (1.46)	17.11 (5.21)	119.45 (15.69)
*p*-value	<0.001^*^	0.106	0.711	<0.001^*^	0.334
*b*-value	0.144	0.109	0.075	3.003	2.101
**Adults**					
Paper	5039.54 (1414.62)	1550.43 (642.99)	8.83 (3.31)	30.46 (7.90)	56.45 (15.63)
Tablet	5872.78 (1823.93)	1741.86 (617.29)	8.51 (3.25)	35.88 (8.93)	59.68 (15.67)
*p*-value	<0.001^*^	0.002^*^	0.031^*^	<0.001^*^	0.010^*^
*b*-value	0.156	0.104	−0.369	6.502	2.982

*The p-value refers to the comparison for Medium (tablet vs. paper). The b-value refers to the regression coefficient of the tablet condition in comparison to paper. The asterisk indicates significant effects below an alpha-level of 0.05*.

The correlation analyses between writing velocity and NIVs confirmed again that these two measures are negatively related for both groups (all *p* < 0.001; second grade τ = −0.36, adults τ = −0.20), showing that a faster velocity is associated with fewer NIVs.

Regarding group differences we found that second graders wrote longer (*p* < 0.001, *b* = 1.125), produced longer in air times (*p* < 0.001, *b* = 0.613), and more pen lifts (*p* < 0.001, *b* = −3.474), wrote slower (*p* < 0.001, *b* = −16.132), and exhibited more NIVs (*p* < 0.001, *b* = 62.577) than adults. There were no significant interactions between the factors Medium and Group.

A quite paradoxical result that we pursued further with additional analyses was the fact that participants showed a longer writing duration and a higher writing velocity on the tablet compared to paper. This result turned out to reflect a difference in letter size between the two writing surfaces. Paired *t*-tests revealed that participants in both groups wrote larger on the tablet (second grade *M*: 1.72 cm, *SD*: 0.38 cm; adults *M*: 1.47 cm, *SD*: 0.29 cm) compared to paper (second grade *M*: 1.36 cm, *SD*: 0.33 *t*_(296)_ = −18.24, *p* < 0.001; adults *M*: 1.20 cm, *SD*: 0.32 cm; *t*_(299)_ = −18.20, *p* < 0.001).

**Figure 6:**
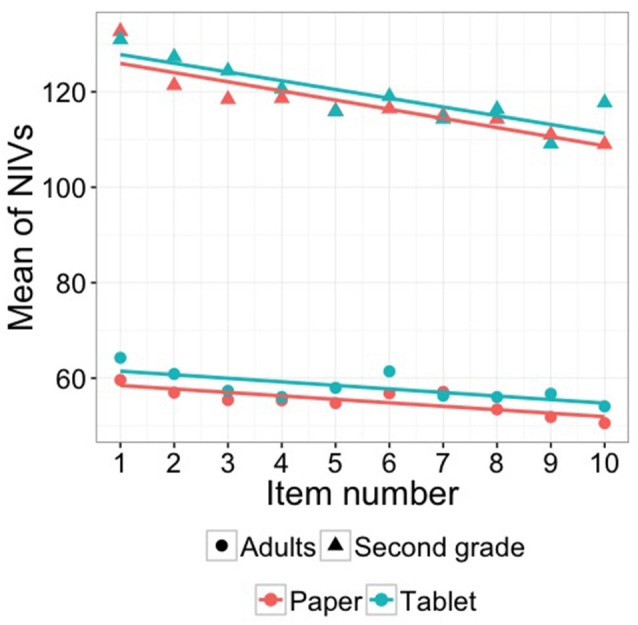
**The mean of the NIVs for each item of “sun and waves” on paper and on the tablet computer (with 95% confidence intervals)**.

We conducted another additional analysis to test if our participants adapted to the unfamiliar and smoother surface of the tablet over time. Therefore, we ran linear mixed-effects models with the NIVs as the dependent variable and item number in increasing order as the independent variable. If participants adapted to the unfamiliar writing surface then the NIVs should decrease over the course of task (writing the phrase 10 times), revealing an increase in automatization and a decrease in the focus on the graphomotor execution of the task. For both media (tablet and paper) the NIVs significantly decreased for both groups (second grade: paper: *p* < 0.001, *b* = –1.969, tablet: *p* < 0.001, *b* = –1.982; adults: paper: *p* < 0.001, *b* = –0.720, tablet: *p* < 0.001, *b* = –0.772) from the first (second grade: paper: *M*: 132.68, *SD*: 22.73; tablet: *M*: 130.95, *SD*: 20.15; adults: paper: *M*: 59.61 *SD*: 17.43; tablet: *M*: 64.26 *SD*: 14.85) to the last item (second grade: paper: *M*: 109.00, *SD*: 12.76; tablet: *M*: 117.73, *SD*: 15.84; adults: paper: *M*: 50.56 *SD*: 14.09; tablet: *M*: 54.08 *SD*: 16.00) of writing repetitively the same phrase. Figure 6 visualizes the decrease in NIVs for both groups and both media.

## Discussion

The present study investigated whether the writing surface (tablet vs. paper) influences the product and the process of writing. In order to identify task-dependent modulations of this influence, we used three tasks to test (1) graphomotor abilities using repetitive patterns, (2) visuomotor abilities, and (3) handwriting abilities. As a second aim we sought to reveal the relationship between the evaluation of handwriting quality and the dynamics of the handwriting process. Thirdly, we wanted to investigate the different levels of handwriting automaticity in three groups (preschoolers, second graders, and adults).

Our results demonstrate important differences between writing on a tablet and writing on paper. Similar to the study by Gerth et al. (2016) the findings are task-dependent and specific to the writing demands of the tasks. We will interpret our results in more detail according to the comparison between writing surfaces (Section Handwriting on the Tablet vs. Paper), the comparison between quality measures and process measures of handwriting (Section Handwriting Product vs. Process) and between-group differences (Section Age-Related Effects of Handwriting Performance).

### Handwriting on the tablet vs. paper

Our evaluation of handwriting quality yielded differences between writing on the tablet and on paper for the three groups. In particular the children groups showed a higher handwriting quality when writing on paper for some of the graphomotor and for both visuomotor tasks. Contrastingly, the adults showed the opposite pattern (better handwriting quality when writing on the tablet) for two of the graphomotor tasks (zigzag lines and staircase pattern). Since children are not automatized in their writing movements, they seem to be challenged most by a decrease in proprioceptive feedback of the writing surface. The adults, however, seem to adapt to the smoother surface quite quickly and effortlessly during the course of the task because they show the better performance on the tablet for the last two tasks in this task battery (zigzag lines and staircase pattern). We can only speculate that adults might have concentrated less on the accurate execution of the task on paper because this writing surface is very familiar to them.

Regarding the handwriting process measures we found a faster writing velocity on the tablet compared to paper for all groups and the majority of tasks. These findings indicate that the pen was sliding faster on the tablet which might have been due to the lower friction of the surface. In order to perform a fluent and regular writing movement, participants had to adapt their graphomotor execution. In our first task—testing graphomotor abilities by copying repetitive pattern—we found significantly faster writing velocity for all tasks and all groups (except for the staircase pattern in the preschoolers). When comparing the results between media for the visuomotor tasks—VMI and MC—we found again that all groups performed the tasks with a higher velocity on the tablet compared to paper (except for the MC in the adults' group). The additional analyses comparing task demands revealed main differences for all writing measures in the children's groups and three handwriting measures for the adults (writing duration, velocity, and NIVs). Apparently the task demands of the MC were higher compared to the VMI because participants had to stay in a predefined writing area. Drawing the attention to the writing process clearly hampers the automaticity of the writing movements and leads to a slower execution (Tucha and Lange, 2005; Tucha et al., 2008). In our third task—probing handwriting—participants copied a phrase of three words for ten times. This task directly tests automatized handwriting movements that are stored in motor programs of experienced writers. We obtained a longer writing duration and a faster writing velocity for both groups on the tablet which is due to the fact that both groups wrote bigger letters on the tablet with a higher velocity. The smoother surface presumably requires a higher graphomotor control to counter the lower proprioceptive feedback of the surface (lower friction). One way to adapt the writing movements is to enlarge the letter size which corroborates findings of previous research (Denier van der Gon and Thuring, 1965; Alamargot and Morin, 2015; Gerth et al., 2016). It is interesting to see that even second graders who are in the middle of handwriting acquisition are already capable of compensating the smoother surface with this adaptation in graphomotor execution. This might reveal that they are relying more on the proprioceptive rather than visual feedback similar to experienced writers, which might reflect that they use the ability of motor anticipation for writing and activate their motor programs quickly and automatically (Kandel and Perret, 2015).

### Handwriting product vs. process

In our study we used two measures for the handwriting assessment—the handwriting quality evaluated by a visual score and the handwriting process measures as a direct measure of the level of automaticity in handwriting. As expected, both measures reflect different dimensions of handwriting task results (similar to results by Fliesser et al., in preparation). The score for the handwriting quality relates to the visual legibility and alignment of words and may be appropriate to test the level of handwriting proficiency of the writer since children are taught to write neatly and copy the given letter as accurately as possible from the teacher or from a book. Our findings show that all groups were able to copy repetitive patterns, geometric forms, and words on both media. The disadvantages of performing the tasks on the tablet are expected since the smoother surface introduces an unfamiliar writing surface with a lower friction that has to be countered with higher graphomotor control of the writing movements. This is also visible in the higher writing velocity (as one of our handwriting process measures) for nearly all our tasks in all groups on the tablet. The velocity, which is negatively related to the NIVs as measure of an automatized and fluent handwriting movement, reflects the participant's ability to coordinate fine muscles to control the graphomotor execution and produce a fluent movement. Hence these process measures seem to refer to the motor component of writing rather than the visual control. Therefore, we believe that only the combination of both measures provides a complete picture of the level of handwriting skills in children and adults: product-oriented handwriting measures reflect the visual control and feedback during writing, whereas process-oriented measures mirror a combination of the graphomotor and visual control.

### Age-related effects of handwriting performance

When comparing the handwriting performance of our three groups—preschoolers, second graders, and adults—we obtained results in the predicted direction for the handwriting quality and the handwriting process measures. The preschoolers who have not received any writing instructions yet produced the lowest handwriting quality, wrote longer, and slower than the other two groups, paused for a longer time, lifted the pen more often and produced more NIVs in all tasks. Since we designed our tasks in such a way that they were suited for preschoolers they could perform them even without proper writing instructions. Nevertheless, their graphomotor execution was clearly at a non-automatized level and particularly the high number of error points for the graphomotor abilities tasks shows that the tasks were quite demanding. In particular, preschoolers lifted the pen more often than adults in all four tasks and they lifted the pen more often than second graders for loops without dots and zigzag lines. Especially zigzag lines who denote diagonal lines are very demanding for preschool children (Lange-Küttner, 1998) and our results seem to indicate that they used more visual control than second graders and adults to correctly copy the zigzag pattern (= longer pauses and more pen lifts). This behavior might suggest a motor anticipation of the upcoming stroke which takes longer for a complex and unfamiliar graphomotor movement (Kandel and Perret, 2014).

Regarding our visuomotor tasks we found that the preschoolers and second graders wrote faster but produced more NIVs than the adults for the MC. When combining these results with the scores of the handwriting quality evaluation we interpret this finding as a speed-accuracy trade-off. Both children groups obtained a lower accuracy score than adults in this task, but they executed the task faster. Hence, our findings indicate that the MC was more demanding for the children. They performed faster (higher velocity), but had to focus their attention stronger on the graphomotor execution (higher NIVs) and were still less accurate. This result is unsurprising since the MC required the participants to stay in a predefined writing space to copy the geometrical forms accurately. Apparently the children had greater difficulties to control the pen on the smoother tablet surface during this task. The combination of visual and graphomotor control without familiar proprioceptive feedback hampered the (automaticity in) writing movements which is similar to studies during which participants had to visually track the pen tip during writing and produced more NIVs (Marquardt et al., 1996; Tucha and Lange, 2005; Tucha et al., 2008; Gerth et al., 2016).

Our handwriting task revealed that the second graders wrote slower, lifted the pen more often, made longer pauses and exhibited more NIVs compared to the adults. Further, both groups compensated the smoother surface of the tablet with an increase in letter size which corroborates findings in previous research (Denier van der Gon and Thuring, 1965; Alamargot and Morin, 2015; Gerth et al., 2016). Our additional analysis testing for a change in the NIVs over all ten items of writing the phrase “Sonne und Wellen” showed that for both groups the NIVs decreased from the first to the last item. Since this task directly depicts handwriting performance it might have been easier for both groups compared to the other two tasks, during which they had to copy patterns, because they write words probably every day. Therefore, we interpret the declining NIVs as a decrease in attention to the writing process and an adaptation of the handwriting movements to the writing surface (even to the smoother tablet).

Apart from main group differences we also found significant interactions between the factors Medium and Group. For the handwriting product evaluation we see a difference in the performance between the adults and the children groups for the zigzag lines and the staircase pattern (see Figure 4). The children produced more error points on the tablet whereas the adults performed worse on paper. When looking more closely at the different categories of the error points we saw that the worse performance of the adults is due to the penalty for lifting the pen while drawing the pattern. Adults lifted the pen more often on paper compared to on the tablet. This suggests that they probably resisted the urge to lift the pen on the tablet presumably because they did not want to risk not to be able to start the new stroke at exactly the same point where they ended the last stroke. For the tablet there was a small gap between the plastic writing surface and the actual screen with the visual feedback of the pen tip. When performing the task on paper there is no gap between the pen tip and the surface, therefore the end point of the previous stroke could be targeted more easily.

The majority of significant interactions between medium and group is due to the fact that the preschoolers show a significant difference between performing the tasks on paper or on the tablet whereas the adults do not show a between-media difference. This result can be interpreted in the light of a difference in experience with the two media. Adults might be more familiar with tablets in general than preschoolers, although this experience could be mostly related to typing on the tablets rather than writing with a pen on the tablet. The lower experience with the tablet as a writing surface is also visible in our data in a higher variability (greater standard deviations) in handwriting performance on the tablet compared to paper. However, all our participants show this higher variance. Therefore, we think that the interactions between media and group in our results rather stem from a different degree in handwriting automaticity of our groups. Especially preschoolers show differences between the two media because they are not automatized in their writing movements and have to counter the low friction of the tablet surface with additional focus on their graphomotor execution. The adults, however, adapt very quickly to the smoother tablet surface because their handwriting movements are stored in the motor programs and they simply need to fine-tune them to counter the lower friction. Apparently this is very difficult for beginning learners. The second graders are somewhere in the middle of their handwriting development. This is also reflected in our results. The second graders show media-differences in the handwriting process measures for the demanding tasks similarly to the preschoolers (e.g., zigzag lines, staircase pattern), but they mostly pattern with the adults' group regarding their handwriting performance.

## Conclusion

The findings of our study provide a first answer to the question whether there are age-related effects in graphomotor execution due to differences in writing surfaces. We found differences between writing on paper compared to the tablet. These differences were partly task-dependent. Generally, we found a higher writing velocity for writing movements on the tablet which indicates that all groups—even the experienced writers—were influenced by the lower friction of the writing surface. Apparently the pen was sliding stronger on the smoother surface of the tablet.

Our results of the between-group analyses revealed that the non-writers (preschoolers), beginning writers (second graders), and the experienced writers (adults) were differently influenced by the two writing surfaces. Especially when the task required a combination of visual and graphomotor control (such as the MC) the children were particularly challenged by the smoother surface of the tablet. Therefore, we doubt that it is recommendable to use tablets in schools for writing acquisition because the smoother surface represents an additional challenge for learners of writing that they have to counter with an increased control of their graphomotor execution. This might lead to a prolongation of handwriting acquisition and possibly increases children's frustration when trying to write most legibly.

Further we do not think that it is wise to simply digitize paper-pencil-versions of a test to obtain measures of the handwriting dynamics. Our results show a task-dependency and differences in task-demands that might lead to unexpected results due to the unfamiliar and smoother surface of a tablet and not due the experimental manipulation itself.

## Author contributions

TD has been part of the Research Group, and is currently at the University of Regensburg. Author contributions are as follows: substantial contributions to the conception and design of the project (all authors); data acquisition (SG), data analysis (SG), and interpretation of data (SG, JF, AK, TD, MF), drafting the manuscript (SG, JF), revising it critically for important intellectual content (all authors).

## Funding

This research was funded by the Land Brandenburg, Germany. We acknowledge the support of the Deutsche Forschungsgemeinschaft and Open Access Publishing Fund of University of Potsdam.

### Conflict of interest statement

The authors declare that the research was conducted in the absence of any commercial or financial relationships that could be construed as a potential conflict of interest.

## Footnotes

^1^We did not run analyses on *in air time* and *number of pen lifts* if *in air time* was 0ms or the *number of pen lifts* was equal to 1.

^2^We used Kendall's tau correlations because the handwriting measures were not normally distributed.

## References

[B1] Abbott R. Berninger V. W. (1993). Structural equation modeling of relationships among developmental skills and writing skills in primary- and intermediate-grade writers. J. Educ. Psychol. 85, 478–508. 10.1037/0022-0663.85.3.478

[B2] Accardo A. P. Genna M. Borean M. (2013). Development, maturation and learning influence on handwriting kinematics. Hum. Mov. Sci. 32, 999–1009. 10.1016/j.humov.2013.08.002 24128883

[B3] Adi-Japha E. Freeman N. H. (2001). Development of differentiation between writing and drawing systems. Dev. Psychol. 37, 101–114. 10.1037/0012-1649.37.1.101 11206425

[B4] Alamargot D. Morin M.-F. (2015). Does handwriting on a tablet screen affect students' graphomotor execution? A comparison between grades two and nine. Hum. Mov. Sci. 44, 32–41. 10.1016/j.humov.2015.08.011 26298215

[B5] Beery K. E. Beery N. A. (2010). Beery VMI, 6th Edn. Bloomington, IL: Pearson.

[B6] Berninger V. W. Abbott R. D. Abbott S. P. Graham S. Richards T. (1998). Writing and reading: connections between language by hand and language by eye. J. Learn. Disabil. 35, 39–56. 10.1177/002221940203500104 15490899

[B7] Berninger V. W. Vaughan K. B. Abbott R. D. Abbott S. P. Woodruff Rogan L. Brooks A. (1997). Treatment of handwriting problems in beginning writers: transfer from handwriting to composition. J. Educ. Psychol. 89, 652–666. 10.1037/0022-0663.89.4.652

[B8] Berninger V. Yates C. Cartwright A. Rutberg J. Remy E. Abbott R. (1992). Lower-level developmental skills in beginning writing. Read. Writ. 4, 257–280. 10.1007/BF01027151

[B9] Bos W. Eickelmann B. Gerick J. (2014). ICILS 2013 auf einen Blick International Computer and Information Literacy Study. Münster: Waxmann.

[B10] Chartrel E. Vinter A. (2006). Rôle des informations visuelles dans la production de lettres cursives chez l'enfant et l'adulte. Annee Psychol. 106, 43–64. 10.4074/S0003503306001047

[B11] Chicu S. O. Ţicău A. Şoitu L. (2014). Training for new technologies. Handwriting with new technologies. Proc. Soc. Behav. Sci. 142, 781–785. 10.1016/j.sbspro.2014.07.616

[B12] Cornhill H. Case-Smith J. (1996). Factors related to good and poor handwriting. Am. J. Occup. Ther. 50, 732–739. 10.5014/ajot.50.9.732 8886192

[B13] Daly C. J. Kelley G. T. Krauss A. (2003). Relationship between visual-motor integration and handwriting skills of children in kindergarten: a modified replication study. Am. J. Occup. Ther. 57, 459–462. 10.5014/ajot.57.4.459 12911088

[B14] Denier van der Gon J. J. Thuring J. P. (1965). The guiding of human writing movements. Kybernetik 2, 145–148.

[B15] Exner C. E. (2010). Evaluation and interventions to develop hand skills, in Occupational Therapy for Children, eds Case-Smith J. O'Brian J. C. (MOSBY, Elsevier), 276–277.

[B16] Feder K. P. Majnemer A. (2007). Handwriting development, competency, and intervention. Dev. Med. 49, 312–317. 10.1111/j.1469-8749.2007.00312.x 17376144

[B17] Fischer K. Wendler M. (1994). Der Schriftsprachenerwerb und die kindliche Entwicklung. Neurowissenschaftliche Grundlagen und praktische Konsequenzen fur eine graphomotorische Forderung. Kindheit Entwicklung 3, 74–83.

[B18] Flower L. Hayes J. R. (1981). A cognitive process theory of writing. Coll. Compos. Commun. 32, 365–387. 10.2307/356600

[B19] Gerth S. Dolk T. Klassert A. Fliesser M. Fischer M. H. Nottbusch G.. (2016). Adapting to the surface: a comparison of handwriting measures when writing on a tablet and on paper. Hum. Mov. Sci. 48, 62–73. 10.1016/j.humov.2016.04.006 27132154

[B20] Gombert J. E. Fayol M. (1992). Writing in preliterate children. Learn. Instr. 2, 23–41. 10.1016/0959-4752(92)90003-5

[B21] Graham S. Weintraub N. (1996). A review of handwriting research: progress and prospects from 1980 to 1994. Educ. Psychol. Rev. 8, 7–87. 10.1007/BF01761831

[B22] Hamstra-Bletz L. Blöte A. W. (1993). A longitudinal study on dysgraphic handwriting in primary school. J. Learn. Disabil. 26, 689–699. 10.1177/002221949302601007 8151209

[B23] Huber R. A. Headrick A. M. (1999). Handwriting Identification: Facts and Fundamentals. Boca Raton, FL: CRC Press.

[B24] Julius M. S. Adi-Japha E. (2015). Learning of a simple grapho-motor task by young children and adults: similar acquisition but age-dependent retention. Front. Psychol. 6:225. 10.3389/fpsyg.2015.00225 25798120 PMC4350392

[B25] Kaiser M.-L. Albaret J.-M. Doudin P.-A. (2009). Relationship between visual-motor integration, eye-hand coordination, and quality of handwriting. J. Occup. Ther. Sch. Early Interv. 2, 87–95. 10.1080/19411240903146228

[B26] Kandel S. Perret C. (2014). How do movements to produce letters become automatic during writing acquisition? Investigating the development of motor anticipation. Int. J. Behav. Dev. 39, 113–120. 10.1177/0165025414557532

[B27] Kandel S. Perret C. (2015). How does the interaction between spelling and motor processes build up during writing acquisition? Cognition 136, 325–336. 10.1016/j.cognition.2014.11.014 25525970

[B28] Lange-Küttner C. (1998). Pressure, velocity, and time in speeded drawing of basic graphic patterns by young children. Percept. Mot. Skills 86, 1299–1310. 10.2466/pms.1998.86.3c.1299 9700806

[B29] Laszlo J. L. Broderick P. (1991). Drawing and handwriting difficulties: reasons for and remediation of dysfunction, in Development of Graphic Skills: Research, Perspectives and Eductional Implications, eds Wann J. Wing A. M. Sovik N. (London: Academic Press), 259–280.

[B30] Latash M. L. (1993). Control of Human Movement. Urbana, IL: Human Kinetics Publishers.

[B31] Maldarelli J. E. Kahrs B. A. Hunt S. C. Lockman J. J. (2015). Development of early handwriting: visual-motor control during letter copying. Dev. Psychol. 51, 879–888. 10.1037/a0039424 26029821 PMC4478098

[B32] Marquardt C. Gentz W. Mai N. (1996). On the role of vision in skilled handwriting, in Handwriting and Drawing Research: Basic and Applied Issues, eds Simner M. L. Leedham C. G. Thomassen A. J. W. M. (Amsterdam: IOS Press), 87–97.

[B33] Marquardt C. Mai N. (1994). A computational procedure for movement analysis in handwriting. J. Neurosci. Methods 52, 39–45. 10.1016/0165-0270(94)90053-1 8090015

[B34] Medwell J. Wray D. (2007). Handwriting: what do we know and what do we need to know? Literacy 41, 10–15. 10.1111/j.1467-9345.2007.00453.x

[B35] Medwell J. Wray D. (2014). Handwriting automaticity: the search for performance thresholds. Lang. Educ. 28, 34–51. 10.1080/09500782.2013.763819

[B36] Meulenbroek R. G. J. Van Galen G. P. (1986). Movement analysis of repetitive writing behaviour of first, second and third grade primary school children, in Graphonomics: Contemporary Research in Handwriting, eds Kao H. S. R. Van Galen G. P. Hoosain R. (North-Holland: Elsevier Science Publishers B.V.), 71–92.

[B37] Meulenbroek R. G. J. Van Galen G. P. (1989). The production of connecting strokes in cursive writing: developing co-articulation in 8 to 12 year-old children, in Computer Recognition and Human Production of Handwriting, eds Plamondon R. Suen C. Y. Simner M. L. (Singapore: World Scientific), 105–118.

[B38] Meulenbroek R. G. Van Galen G. P. (1990). Perceptual-motor complexity of printed and cursive letters. J. Exp. Educ. 58, 95–110. 10.1080/00220973.1990.10806527

[B39] Pinheiro J. Bates D. DebRoy S. Sarkar D. Team R. C. (2014). nlme: Linear and Nonlinear Mixed Effects Models. R Packag. version 3.1-118. Available online at: https://cran.r-project.org/web/packages/nlme/index.html

[B40] Pontart V. Bidet-Ildei C. Lambert E. Morisset P. Flouret L. Alamargot D. (2013). Influence of handwriting skills during spelling in primary and lower secondary grades. Front. Psychol. 4:818. 10.3389/fpsyg.2013.00818 24204357 PMC3817363

[B41] R Development Core Team (2013). A Lang. Environ. Stat. Comput. 55, 275–286. Available online at: http://www.r-project.org

[B42] Read J. C. MacFarlane S. Horton M. (2005). The usability of handwriting recognition for writing in the primary classroom in People and Computers XVIII - Design for Life, eds Fincher S. Markopoulos P. Moore D. Ruddle R. (London: Springer), 135–150.

[B43] Reisman J. E. (1999). Minnesota Handwriting Assessment. San Antonio, TX: Psychological Corporation.

[B44] Rosenblum S. Dvorkin A. Y. Weiss P. L. (2006). Automatic segmentation as a tool for examining the handwriting process of children with dysgraphic and proficient handwriting. Hum. Mov. Sci. 25, 608–621. 10.1016/j.humov.2006.07.005 17011656

[B45] Rosenblum S. Weiss P. L. Parush S. (2003). Product and process evaluation of handwriting difficulties. Educ. Psychol. Rev. 15, 41–81. 10.1023/A:1021371425220

[B46] Sumner E. Connelly V. Barnett A. L. (2013). Children with dyslexia are slow writers because they pause more often and not because they are slow at handwriting execution. Read. Writ. 26, 991–1008. 10.1007/s11145-012-9403-6

[B47] Sumner E. Connelly V. Barnett A. L. (2014). The influence of spelling ability on handwriting production: children with and without dyslexia. J. Exp. Psychol. Learn. Mem. Cogn. 40, 1441–1447. 10.1037/a0035785 24548322

[B48] Tseng M. H. Chow S. M. K. (2000). Perceptual-motor function of school-age children with slow handwriting speed. Am. J. Occup. Ther. 54, 83–88. 10.5014/ajot.54.1.83 10686631

[B49] Tucha O. Lange K. W. (2005). The effect of conscious control on handwriting in children with attention deficit hyperactivity disorder. J. Atten. Disord. 9, 323–332. 10.1177/1087054705279994 16371678

[B50] Tucha O. Tucha L. Lange K. W. (2008). Graphonomics, automaticity and handwriting assessment. Literacy 42, 145–155. 10.1111/j.1741-4369.2008.00494.x

[B51] Van Galen G. P. (1991). Handwriting: issues for a psychomotor theory. Hum. Mov. Sci. 10, 165–191. 10.1016/0167-9457(91)90003-G

[B52] Volman M. J. M. van Schendel B. M. Jongmans M. J. (2006). Handwriting difficulties in primary school children: a search for underlying mechanisms. Am. J. Occup. Ther. 60, 451–460. 10.5014/ajot.60.4.451 16915876

[B53] Weil M. J. Cunningham-Amundson S. J. (1994). Relationship between visuomotor and handwriting skills of children in kindergarten. Am. J. Occup. Ther. 48, 982–988. 10.5014/ajot.48.11.982 7840134

[B54] Wollscheid S. Sjaastad J. Tømte C. (2016). The impact of digital devices vs. Pen(cil) and paper on primary school students' writing skills - A research review. Comput. Educ. 95, 19–35. 10.1016/j.compedu.2015.12.001

